# Immune Tolerance in the Oral Mucosa

**DOI:** 10.3390/ijms222212149

**Published:** 2021-11-10

**Authors:** Hector F. Pelaez-Prestel, Jose L. Sanchez-Trincado, Esther M. Lafuente, Pedro A. Reche

**Affiliations:** Laboratory of Immunomedicine, Faculty of Medicine, University Complutense of Madrid, Ave Complutense S/N, 28040 Madrid, Spain; hpelaez@ucm.es (H.F.P.-P.); josels07@ucm.es (J.L.S.-T.)

**Keywords:** oral, mucosa, tolerance, T cell, dendritic cell, epithelial cell

## Abstract

The oral mucosa is a site of intense immune activity, where a large variety of immune cells meet to provide a first line of defense against pathogenic organisms. Interestingly, the oral mucosa is exposed to a plethora of antigens from food and commensal bacteria that must be tolerated. The mechanisms that enable this tolerance are not yet fully defined. Many works have focused on active immune mechanisms involving dendritic and regulatory T cells. However, epithelial cells also make a major contribution to tolerance by influencing both innate and adaptive immunity. Therefore, the tolerogenic mechanisms concurring in the oral mucosa are intertwined. Here, we review them systematically, paying special attention to the role of oral epithelial cells.

## 1. Anatomy of Oral Mucosa and Associated Lymphoid Tissues

**Figure 1:**
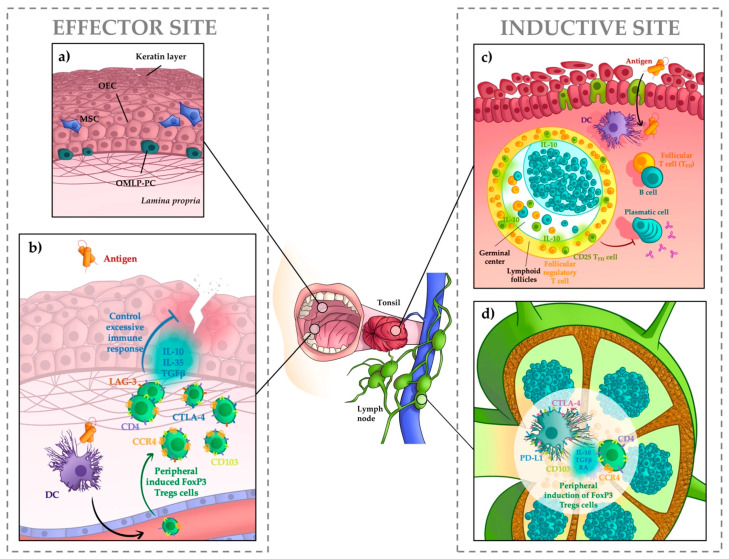
The oral mucosa and associated lymphoid structures. The oral mucosa is lined by stratified squamous epithelia of varying thickness that can be topped by a keratin layer. Some areas related with mastication (**a**) are thick and highly keratinized, while other zones, such as the lining mucosa (**b**), are thinner and feature no keratin layer. Oral epithelial cells (OECs) are derived from local progenitor cells such as oral mucosal lamina propria-progenitor cells (OMLP-PCs). Resident mesenchymal stem cells (MSC) can also differentiate to epithelial cells as well as many other cell types. Resident dendritic cells (DC) can capture these antigens and migrate to secondary lymphoid tissues, including the tonsils (**c**) and proximal lymph nodes (**d**), where adaptive immune responses are elicited (inductive sites). CD103^+^ CCR4^+^ Tregs are induced in draining secondary lymphoid tissues (**d**) and migrate to the effector site, where they suppress immune responses when needed via CTLA-4 and LAG-3 expression and IL-10 and TGFβ production (**b**). The tonsils are exposed to a constant bombardment of antigens from innocuous sources, such as food, that need to be tolerated. Regulatory follicular T cells (T_FR_) as well as CD25^+^ T_FH_ cells also control GC size and class switching by releasing IL-10 (**c**).

The oral cavity is the entry to the gastrointestinal tract, participating in mastication and the tasting of food. Therefore, the structure and anatomy of the oral cavity are quite unique, as Figure 1 shows. The oral mucosa is the membranous tissue lining the inside of the mouth, consisting of oral epithelial cells and a layer of connective tissue underneath, known as lamina propria. Unlike the gut mucosa, which only includes a single layer of columnar epithelial cells, the oral mucosa comprises a stratified epithelium, consisting of increasingly keratinized layers of squamous epithelial cells piling over the lamina propria. The keratinization degree is not homogeneous throughout the oral mucosa, varying depending on their anatomic localization and function. For instance, the masticatory epithelium (the palate) which could be easily injured through chewing, is protected by a thick keratin layer, resembling the skin, which is likely to make antigen entry difficult [1]. By contrast, other sites, such as the lining mucosa (the internal part of the cheek, inside of the lips, floor of the mouth or soft palate) or the sublingual area, feature no keratin layer [2,3]. Besides, the oral cavity houses two more elements of complexity, the teeth and the tongue, with their own associated mucosa. On the one hand, the tongue is a heterogeneous and complex tissue, displaying sensing, motor, and barrier functions. Therefore, many cell types constitute this organ, including a specialized epithelium that incorporates the taste buds [4]. On the other hand, the gingival mucosa that surrounds the dental piece, must cope with constant environmental stimuli, since the combination of the physical damage of mastication and dental bacteria turn this area into one of the most vulnerable places of the oral cavity [5].

The oral mucosa features with various mucosa-associated lymphoid tissues that control immune responses. They are part of Waldeyer’s ring, which is composed by the pharyngeal tonsil placed at the roof of the nasopharynx, the tubal tonsils located on the lateral wall of the nasopharynx, the palatine tonsils located on the left and right sides at the back of the oropharynx, and the lingual tonsils at the back of tongue. These lymphoid structures are in intimate contact with the epithelium [6]. In addition, more than 300 out of the 800 lymph nodes in the human body are in the neck and head, draining the mucosal tissue [7]. In these lymphoid tissues, immune responses are elicited. 

## 2. Immune Responses in the Oral Mucosa

The structure of the oral cavity reflects its important role in the process of digestion. However, the oral cavity is also a site of intense immune activity, where many pathogenic organisms are first encountered and fought. The elicitation of defense immune responses involves inductive and effector sites. Inductive sites are where most lymphocytes are activated and expanded upon antigen stimulation, while the effector sites are where activated lymphocytes migrate and relocate to mediate immune responses. In the oral mucosa, the inductive sites are the tonsils and proximal lymph nodes, while the effector sites include the epithelium, the lamina propria, and the salivary glands [8]. 

Antigens captured at the oral mucosa are recognized by lymphocytes in proximal associated lymphoid tissues. At the base of the tonsillar crypts (invaginations of the tonsillar stratified epithelium that greatly increase the epithelium surface), there are specialized micropore (M) cells, which facilitate the transport of these antigens into the tonsils. There, antigens are taken up by dendritic cells (DCs) and presented to T helper cells and B cells, which form the germinal centers of the tonsil. In the germinal centers, the production of antibodies takes place, initiating the adaptive immune response [9,10]. In addition, resident DCs likely capture antigens at the non-keratinized parts of the oral mucosa and migrate to the tonsils or proximal lymph nodes to initiate immune responses there. Subsequently, B and T cells migrate to the effector sites (i.e., the epithelium, or, in the case of B cells, different secretory structures such as immunoglobulin-producing plasma cells) [11]. This process is represented in Figure 1. 

However, antigens alone cannot drive defensive immune response, and there must be additional danger signals. In fact, the tonsils continuously receive antigens from food and resident bacteria without inducing any inflammatory responses. Therefore, the oral mucosa appears to be in a default state of tolerance that is only occasionally broken in the presence of certain danger signals. Typically, regulatory T cells are considered a key player mediating this tolerance, as we demonstrate below. 

## 3. Tolerance Mediated by Regulatory T Cells

Typically, CD4^+^ T are considered as the immune response linchpin. This is due to their unique ability to polarize immune responses, mainly to tolerance or inflammation. A subset of CD4^+^ T cells of special relevance in controlling immune responses are regulatory T (Treg) cells. There are several types of T cells with regulatory activity, but the most important and numerous group of Treg cells in the oral mucosa consists of CD4^+^ T cells expressing CD25 and the master transcriptional factor FoxP3 [12]. Treg cells are characterized by their secretion of the cytokine IL-10, along with TGFβ and IL-35 [13]. These cytokines contribute to immune suppression, inhibiting the synthesis and secretion of pro-inflammatory factors, downregulating the expression of MHCII and co-stimulatory molecules, and suppressing T cell proliferation [14,15,16]. In addition, Tregs cells can also modulate DC function by expressing molecules, such as the Cytotoxic T-Lymphocyte Antigen 4 (CTLA-4), that bind to CD80/CD86, competing for CD28 co-stimulation or lymphocyte activation gene 3 (LAG-3/CD223) that binds MHCII, interfering with the antigen presentation function [17,18]. Foxp3+ Treg cells are either generated in the thymus (tTregs, also known as natural nTregs) or induced in peripheral tissues (known as peripheral pTreg, or inducible iTreg). tTreg cells recognize self-antigens and are thought to prevent autoimmune reactions, whereas pTreg cells are induced in the periphery under exposure to transforming growth factor (TGF) β to maintain immune responses under control [19]. No reliable phenotypical marker exists to discriminate between pTregs and tTregs, except for some activation markers, such as Neuropilin-1 (Nrp-1), which are more abundant in tTregs [20] and are found in lower amounts in oral Treg cells [21], suggesting the peripherally induced tissue origin of oral Foxp3+ Treg cells. Interestingly, these oral Treg cells express large amounts of CD103 [12], increasing the regulatory power of this subset [22]. Treg cells can control immune response in both effector and inductive sites, as represented in Figure 1. Foxp3+ Treg cells are activated in lymphoid structures under inflammatory conditions and then recruited to effector site by specific combinations of chemotactic molecules [23,24]. The CCL22-CCR4 axis seems to be particularly relevant to the recruitment of Tregs cells, as shown in mice periodontitis models [25]. Subsequently, Tregs limit the immune response in the epithelium, avoiding excessive inflammation, through the mechanisms previously explained. 

Tregs regulate antibody production in the secondary lymphoid structures (inductive sites) promoted by B-cell lymphoma 6 protein+ (BCL6) helper follicular T (TFH) cells. TFH cells interact with B cells at the borders between T cell zones and B cell follicles, driving B cells to differentiate into long-lived plasma cells and memory B cells, after repeated cycles of division and mutation within germinal centers (GCs) [26]. However, this process must be regulated to avoid the production of undesired antibodies. Thereby, there is a population of regulatory cells known as follicular regulatory T cells (TFR), composed of FoxP3 and CD25-expressing tTregs and pTregs cells. TFR regulates the proliferation and cytokine secretion of TFH cells, thus modifying humoral responses at different levels (GC size; selection of antigen-specific B-cell clones; modulation of class switch and affinity maturation) [27]. Moreover, a subset of IL-10 producing TFH cells has recently been reported as being present in the tonsils, which, despite its being Foxp3^–^, can also suppress other TFH cells and dampen class switching [28]. 

In summary, Treg cells are key to maintaining immune homeostasis and controlling immune responses in the oral mucosa and associated lymphoid tissue. However, Treg presence and genesis in the oral mucosa requires the intervention of dendritic cells.

## 4. Tolerance Mediated by Dendritic Cells

Dendritic cells (DCs) are a highly heterogeneous population that reside in a quiescent state in peripheral or lymphoid tissues, acting as the sentinel cells of the immune system. In brief, two different subsets of DCs inhabit the oral mucosa in humans: langerin (CD207)-expressing DCs, known as Langerhans cells (LCs), which are embedded in the oral epithelium; and conventional DCs (cDCs) which are found close to the lamina propria. Different subsets of cDC are defined based on the expression of several markers (CD11b, CD11c, Ep-CAM, SIRPα, XCR1, CD207, CD103,) but their specific contribution to mucosal immunity remains uncertain. Moreover, DC subset populations vary throughout the oral mucosa. For instance, a major population of CD11b- and CD103+ DCs is present at the lining mucosa, while these DCs are more limited at the gingival mucosa. CD103 expression is induced by local production of the cytokines Csf-2 (GM-CSF) and Flt3L, which induces Batf3, Id2, and IRF8 transcriptional factors. The expression of this protein confers migratory properties upon DCs, determining their homing and settling on the mucosa; this seems to be related to regulatory properties [29,30]. The source of resident DCs is somewhat uncertain, although some can differentiate in the bone marrow from immature myeloid cells (IMCs). IMCs and myeloid progenitor cells constitute a unique population, known as myeloid-derived suppressor cells (MDSCs). MDSCs possess a strong immunomodulatory capacity, being recruited during inflammatory/infectious processes [31]. In the oral mucosa, MDSCs have mainly been studied in relation to oral squamous cell carcinomas, as they facilitate cancer growth and immune evasion by suppressing T cells [32,33,34]. MDSCs are related to resident macrophages and can actually differentiate into macrophages [31]. The distinction between macrophages and DCs is not trivial and in fact it is a matter of intense debate [35,36]. Resident macrophages are present at the oral mucosa and their numbers increase during inflammation and disease. Macrophages can acquire distinct phenotypes, much like DCs, including pro-inflammatory M1 macrophages involved in host defense and regulatory or anti-inflammatory M2 macrophages [37].

DCs bridge innate and adaptive immunity by capturing antigens and eliciting antigen-specific immune responses. However, DCs in the oral mucosa are constantly exposed to commensal microbes and food antigens, without leading to the generation of undesired immune responses. This is because oral DCs tend to be tolerogenic. Three aspects define tolerogenic DCs: displaying an immature or semi-mature phenotype defined by low expression of co-stimulatory (e.g., CD80, CD83, CD86) and MHC II molecules on their surface (i); releasing anti-inflammatory cytokines, such as IL-10 or TGFβ (ii); and expressing inhibitory surface molecules, such as PD-L1, PD-L2 or CTLA-4 (iii) [38]. Many studies have proven that oral DCs do in fact possess these properties. For instance, the expression of the CD83 maturation marker is decreased in oral LCs in comparison with the skin LC [39]. Ex vivo, a partial maturation of oral LCs with tolerogenic properties under allergen application was reported, a phenomenon that was not detected in epidermal DCs under similar conditions [40]. In addition, tolerogenic properties are induced in oral LCs upon the binding of monophosphoryl lipid A to TLR4, a common ligand used to activate DCs [41]. Tolerogenic DCs can promote tolerance by inducing pTreg generation. For example, murine oral CD103- CD11+ cDCs showed the capacity to differentiate naive CD4+ T cells into FoxP3 Treg cells by releasing retinoic acid and TFGβ [42]. 

In sum, DCs are sentinel cells able to sense danger, and yet in oral mucosa they are particularly resilient to panic, despite the presence of resident bacteria. Mounting evidence indicates that the environment allows oral epithelial cells to keep DCs in a tolerogenic state. 

## 5. Tolerance Begins with Oral Epithelial Cells

Currently, the epithelium is thought to be the mainstay of immune response, articulating tolerance against innocuous antigens in particular. The oral mucosa is no exception. Oral epithelial cells (OECs) constitute a physical barrier between the inner and outer environment. The integrity of this barrier is maintained thanks to tight junctions, which bind neighboring cells and regulate the passage of small molecules through the paracellular pathway [43]. Arguably, OECs are also components of the immune system, being the first cells to sense danger of any kind. They are constantly interacting with a plethora of microbes, mainly bacteria-forming biofilms in oral surfaces. Toll-like receptors (TLRs) and NOD-like receptors are known to be essential in sensing the environment [44]. OECs express TLR2, TLR3, TLR4, NOD1, and NOD2 [45,46], as well as the inflammasome NLRP3 [47]. In this way, OECs can sense and respond to the environment by releasing immunomodulatory factors and by recruiting and engaging other immune cells. Depending on the signals, these two actions guide and determine the type of immune response. 

### 5.1. Soluble Molecules

Among the soluble immunomodulatory factors secreted by OECs, cytokines are the most extensively studied. Several works using cell lines derived from oral epithelial carcinomas have demonstrated that OECs can respond to bacterial stimuli releasing different cytokines. For instance, the OEC lines H413 and TR146 upregulate IL-6 and IL-8 expression when they are stimulated with MV130 (a mixture of inactivated whole cell Gram+ and Gram− bacteria frequently found in the upper respiratory mucosa) [48]. Mirroring these results, the immortalized human OEC line OKF6-TERT2 expresses CXCL3, CXCL1, IL-1, IL-6, colony-stimulating factor-2 (CSF-2), and TNF-α, which are upregulated after incubation with bacterial biofilms [49]. By contrast, primary gingival epithelial cells did not enhance IL-8 production upon stimulation with lipopolysaccharide, lipoteichoic acid and peptidoglycan, while colonic epithelial cells did [50]. In addition, stimulation with MV130 of primary OECs did not have a major impact on the production of IL-6 and IL-8 [48]. The differential response of primary and tumoral OEC to bacterial stimuli can be inferred. Normal primary OECs are in constant interaction with bacteria and are likely desensitized in order to prevent excessive and unnecessary immune responses. 

There is evidence that DCs’ fate depends on the environment created by the epithelial cells [51,52]. The epithelium provides signals to resident DCs, teaching them to be tolerogenic. In such a scenario, resident DCs can orchestrate tolerance towards commensal species through their interaction with T cells [51,53]. Human intestinal epithelial cells (IECs) release retinoic acid and TGFβ, which induces tolerogenic CD103+ DCs, which in turn drive the development of regulatory T (Treg) cells [54]. IECs also secrete thymic stromal lymphopoietin (TSLP), which conditions DCs towards inflammation or tolerance, depending on its concentration [53]. Moreover, it has been shown that excess production of TSLP by epithelial cells in response to external stimuli drives and fuels many pathological Th2 inflammatory responses [55,56,57]. In fact, airborne particles and antigens can trigger the production of cytokines, such as TLSP, through epithelial cells, leading to allergy and asthma. Interestingly, anti-TSLP therapy with blocking antibodies represents a promising new treatment against allergic asthma [58]. 

**Figure 2:**
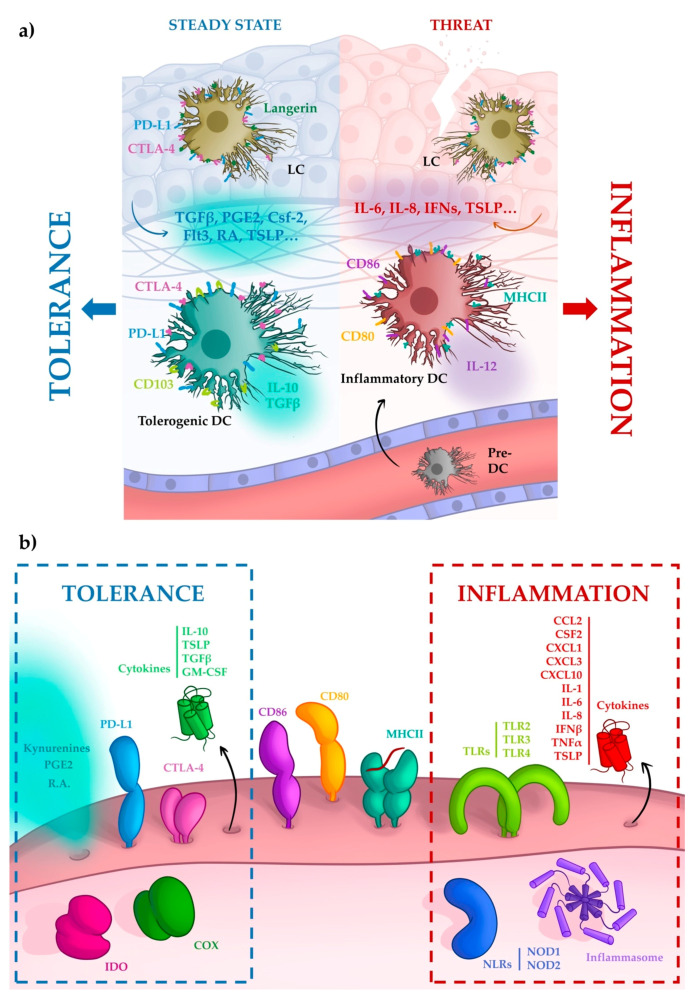
Immune modulation by OECs. (**a**) The fate of the DCs depends on the environment. Under homeostatic conditions, DCs exhibit a tolerogenic phenotype imprinted by surroundings OECs. Low expression of co-stimulatory molecules, high expression of inhibitory molecules and release of tolerogenic cytokines characterize tolerogonic DCs. Moreover, these cells can induce pTreg differentiation. However, threatening conditions, mainly the invasion of the mucosa by pathogenic species may change this environment, and freshly recruited DCs or pre-DCs (immature DCs) develop an inflammatory phenotype able to promote a non-tolerogenic immune response. (**b**) Molecular immunomodulatory arsenal of oral epithelial cells (OECs). OECs express different innate receptors, such as TLRs, NLRs, or inflammasome components that allow them to sense the environment and act in response to pathogens or damage. In addition, OECs modulate the immune response by secreting soluble factors, such as cytokines, prostaglandins, and kynurenines, and by expressing proteins at the cell surface that allow direct interaction with other immune cells, as indicated in Section 5.

When the integrity of the cell barrier is threatened by pathogens, epithelial cells send signals, leading to the recruitment of new pro-inflammatory DCs that elicit defensive immune responses [51]. In the oral mucosa, OECs may also induce tolerogenic properties on oral DCs. In vitro, primary OECs, as well as the OEC lines H413 and TR146, induce partial maturation on MV130-madurated monocyte derived DCs, downregulating the expression of CD80, CD86, and HLA-DR [48]. Overall, this evidence leads us to one conclusion (depicted in Figure 2a): resident oral DCs, either LCs or cDCs, are conditioned by OECs, inducing tolerance regardless of the constant antigenic stimulation they are exposed. However, this tolerance can be broken by a pathogenic insult to the integrity of the oral mucosa. In this scenario, new DCs infiltrate the tissue directed by signals provided by OECs and resident immune cells, promoting the right immune response in the inductive sites. Likewise, OECs can also modulate the immune response of macrophages. To maintain a state of tolerance in the oral mucosa, the balance of macrophage subsets should be tipped towards the M2 phenotype. Thus, recent evidence has suggested the essential role of oral and gingival mesenchymal stem cells (MSC) in re-polarizing macrophages from M1 to M2 through the secretion of IL6, IL-10, GM-CFS, and PGE2 [59].

In the oral mucosa, crosstalk can occur between microbes and immune cells. Oral bacteria such as those in the gut produce great quantities of butyrate. While butyrate plays a beneficial role in the gut mucosa by inhibiting the unneeded immune responses, this metabolite is known to promote periodontitis, since it induces the death of OECs [60]. *Porphyromonas gingivalis*, considered as an opportunistic pathogen, is closely related with this disease, since it also able to persist intracellularly through a process that involves CD73 in the host [61]. When the integrity of the oral mucosa is compromised, wound healing is promoted by oral mucosal lamina propria-progenitor cells (OMLP-PCs). However, OMLP-PCs not only contribute to the maintenance of the oral epithelium, but can also secrete osteoprotegerin (OPG) and haptoglobin (Hp), which are two proteins that produce antibacterial activity [62]. In addition, as the oral epithelium is susceptible to viral infections, OECs also need to respond to viruses. To mimic viral infection, many authors utilize poly(I:C), a synthetic analog of viral double-stranded RNA. Oral squamous cell carcinoma can be robustly stimulated by poly(I:C) upregulating the expression of IL-6, TNFα, IL-8, vascular endothelial growth factor (VEGF), IL-1β, and monocyte chemoattractant protein 1 (MCP-1) (CCL2) [46]. Similarly, we obtained data indicating that the TR146 OEC line stimulated with poly(I:C) also increases the expression of IL-8, IL-6, interferon (IFN)-β, and interferon gamma-induced protein-10 (IP-10, CXCL10). TR146 cells can suppress T cell responses in vitro, but such suppression is partially prevented by incubating these OECs with poly(I:C) (manuscript in preparation). Due to the recent pandemic, how the oral mucosa respond to viruses has become a promising field of study. For instance, SARS-CoV-2 can infect OECs, facilitating viral transmission through respiratory and fecal-oral routes [63]. 

In addition to cytokines, OECs release soluble molecules that could potentially contribute to maintaining immune tolerance and tissue homeostasis. OECs express the enzymes cyclooxygenases (COXs) and indoleamine 2,3-dioxygenases (IDO), which are responsible for the synthesis of the immunomodulatory molecules PGE2 and kynurenines, respectively [64,65]. In fact, OECs secrete PGE2 and contribute to the maintenance of its concentration in saliva under physiological conditions (100 pg/mL) [65,66]. PGE2 is the most abundant prostanoid in the human body, and its pro-inflammatory effect is well known. For decades, nonsteroidal anti-inflammatory drugs (NSAIDs), such as aspirin or indomethacin, have been used as an anti-inflammatory strategy, inhibiting PGE2 synthesis by competing with arachidonic acid for the active sites of both COX enzymes [67]. However, PGE2 can also exert homeostatic and anti-inflammatory effects. For instance, the gastric mucosa produces PGE2 via COX-1 in order to maintain its own integrity; this may explain why the inhibition of PGE2 synthesis with classical nonselective NSAIDs causes significant adverse gastrointestinal effects [68]. PGE2 may play a similar role in the oral mucosa. PGE2 participates in the induction of tolerogenic DCs, as it dampens the secretion of several pro-inflammatory cytokines (TNFα, IL-6, and IL-12) and increases the production of IL-10 in LPS- or CD40L- stimulated DCs [69,70]. In fact, mesenchymal stem cells (MSC), which are located preferentially in the gingival mucosa, as they are related with dental tissue development [71], also express PGE2, which inhibit DCs maturation and activation [72]. Moreover, data indicate that PGE2 released by OECs suppress the local activation of T cell, potentially contributing to protect the integrity of the oral mucosa from undesired responses (manuscript in preparation). Epithelial cells, such as those from the airway epithelium, express IDO, which is upregulated after stimulation with IFNγ [73]. IDO catalyzes the degradation of tryptophan and suppresses T cells by inducing apoptosis and by promoting tolerance through the activation of the aryl-hydrocarbon receptor [74]. Similarly, OMLP-PCs as well as MSCs suppress T cell responses, mainly by expressing this enzyme [75,76].

### 5.2. Surface Molecules

Beyond soluble mediators, OECs can also interact directly with other immune cells, particularly T cells, thanks to the expression of both, suppressing and activating membrane proteins, as well as major histocompatibility complex (MHC) molecules. Regarding the inhibitory proteins, PD-L1 (programmed death-ligand 1) appears to play an important role in the regulation of cell-mediated immune response [77]. The binding of PD-1 and PD-L1 induces the functional anergy and/or apoptosis of effector T cells, promoting tolerance [78]. It has been proposed that this mechanism maintains the eye as an immune-privileged organ, as iris pigment epithelial cells express PD-L1 and suppress T cells activation via PD-1/PD-L1 interaction [79]. Epithelial cells from the renal tubular also express PD-L1 and suppress T cell cytokine synthesis through this same mechanism [80]. In the oral mucosa, this protein is widely expressed. Oral cancer cells, human gingival keratinocytes, and primary oral cells express PD-L1 and this expression can be upregulated by membrane proteins of *P. gingivalis*, a bacteria implicated in periodontal diseases [81,82]. In addition, MSCs also express PD-L1 and PD-L2, suppressing T cell function through this mechanism [83]. Another classical inhibitory molecule is CTLA-4 (cytotoxic T-lymphocyte-associated protein 4), which inhibits costimulatory signal form CD28-CD80/CD86 interaction negatively regulating T cell activation [84]. CTLA-4 was originally defined as a T-lymphocyte antigen, and its expression in other cell types was overlooked for many years. However, a recent study demonstrated that stromal cells and reticular epithelial cells from human tonsils express this protein too, suggesting a broader effect of CTLA-4 on immune regulation and tolerance [85]. Interestingly, neoplastic characters of head and neck cancers (HNSCC) seem to mimic the molecular profile of epithelial cells. In fact, carcinomas (tumors derived from epithelial cells) are the most common type of cancers, perhaps because they exploit the intrinsic ability of epithelial cells to suppress the immune response and scape immune surveillance. They express PDL-1 and secrete TGF- β, PGE2, and IL10, which can promote immunosuppression [86,87,88]. The tumor microenvironment also includes many cells that demonstrate immunosuppressive activity, such as Treg cells, MDSCs, tumor-associated macrophages, tumor-associated neutrophils, and cancer-associated fibroblasts. All these cells enrich the tumor microenvironment with growth factors, hormones, and metalloproteases that promote epithelial-to-mesenchymal transition, cancer progression, and metastasis [89,90].

Finally, several studies have demonstrated that epithelial cells from various sources (esophagus, intestine and colon) can work as non-professional antigen-presenting cells (APCs) and modulate the immune response [91,92,93]. Normal colonic and biliary epithelial cells express MHC II and can directly suppress T cell function despite expressing CD80/CD86 co-stimulatory molecules [94,95]. Han et al. demonstrated that primary-like HOK-18A oral cells increase their expression of costimulatory molecules CD80, CD86, and MHC II in response to pathogenic bacteria in the oral mucosa [96]. However, only MHCII expression was induced on IFNγ-treated TR146, H413, and primary OECs [48]. Similarly, normal oral keratinocytes and other oral cell lines, such as H103 and H157 cells, do not express MHCII molecules constitutively, but they are significantly induced after treatment with IFNγ [97]. Thus, OECs are clearly ready to crosstalk with effector CD4 T cells and likely quench their activity. All these mechanisms are summarized in Figure 2b. 

In conclusion, these mechanisms challenge the traditional view of epithelial cells as passive spectators in the mucosa, suggesting that they are instead actively implicated in immune regulation. OECs are well aware of their surroundings and can promote defensive immune responses. However, OECs inherently create a constitutive tolerogenic environment that maintains immune homeostasis. 

## 6. Concluding Remarks

**Figure 3:**
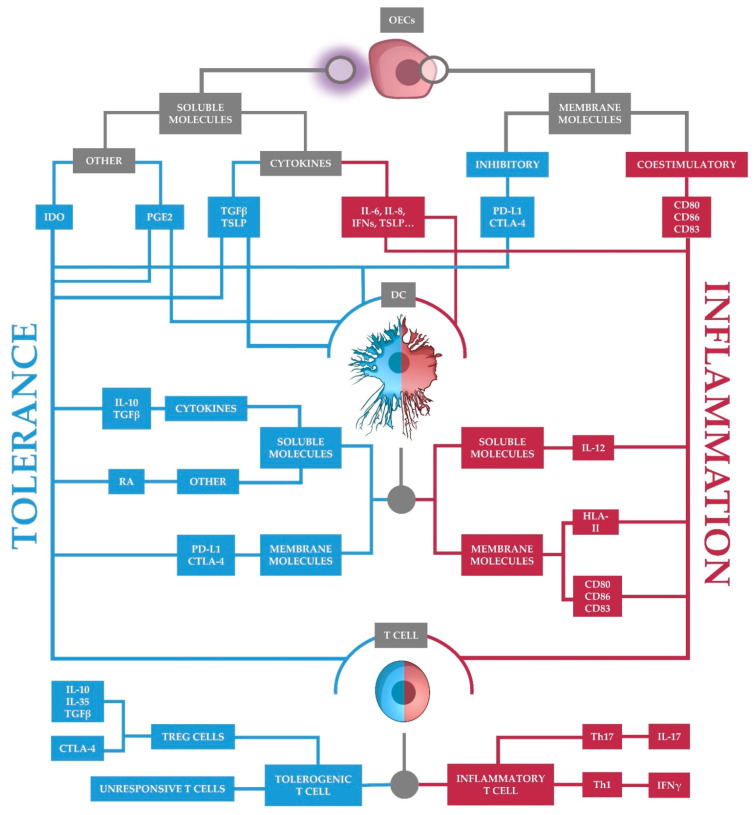
Mechanisms of immune tolerance in the oral mucosa. OECs secrete soluble molecules and express surface proteins that modulate immune responses. They condition DCs to become tolerogenic (blue), which promotes Treg differentiation, but can also promote the recruitment of inflammatory DCs (red). OECs also condition T cell responses by suppressing T cell responses (blue).

The oral mucosa is a site of intense immune activity due to its constant interaction with the environment. Typically, T cells were defined as the main orchestrators of the immune response. Tregs in the mucosa appear to play a key role in suppressing immune responses. However, many other cells contribute to the generation of a tolerogenic environment in the mucosa. Thus, the oral mucosa is populated with tolerogenic DCs that have little capacity to activate T cells but promote regulatory T cell differentiation. In addition, the tolerogenic phenotype of DCs is induced by signals from epithelial cells. In fact, OECs generate a constitutively suppressive environment for other immune cells that helps to maintain immune tolerance. Nonetheless, OECs can also turn the tide of the immune response, allowing inflammatory reactions when they are needed. Therefore, the contribution of oral epithelial cells to oral tolerance is on a par with that of specialized immune cells (Figure 3). In other words, it appears that the tolerance in the oral mucosa is activated by both.

## Data Availability

Not applicable.
